# Air-Stable Na_*x*_TMO_2_ Cathodes for Sodium Storage

**DOI:** 10.3389/fchem.2019.00335

**Published:** 2019-05-14

**Authors:** Yi Zhang, Renyuan Zhang, Yunhui Huang

**Affiliations:** Institute of New Energy for Vehicles, School of Materials Science and Engineering, Tongji University, Shanghai, China

**Keywords:** layered transition metal oxides, air-stable, cathode, sodium-ion battery, water insertion, H ion exchange

## Abstract

Sodium-ion batteries are considered to be the most promising alternative to lithium-ion batteries for large-scale stationary energy storage applications due to the abundant sodium resource in the Earth' crust and as a result, relatively low cost. Sodium layered transition metal oxides (Na_*x*_TMO_2_) are proper Na-ion cathode materials because of low cost and high theoretical capacity. Currently most researchers focus on the improvement of electrochemical performance such as high rate capability and long cycling stability. However, for Na_*x*_TMO_2_, the structure stability against humid atmosphere is essentially important since most of them are instable in air, which is not favorable for practical application. Here we provide a comprehensive review of recent progresses on air-stable Na_*x*_TMO_2_ oxides. Several effective strategies are discussed, and further investigations on the air-stable cathodes are prospected.

## Introduction

The growing demand for large-scale energy storage applications has driven the research interest into new energy storage systems with low cost. Although lithium-ion battery (LIB) can deliver high energy and power density, the limited resource and the rising cost of lithium may restrict their application in grid scale energy storage. Recently, sodium-ion battery (SIB), which owns a similar chemical storage mechanism to LIB, has been rapidly developed as a complementary technology. As the second lightest alkali metal, sodium resource is inexpensive and almost globally available. The common abundant sodium salt such as Na_2_SO_4_, NaCl, and Na_2_CO_3_ could be obtained from marine or mineral. In addition, copper foil can be replaced by cheaper aluminum foil for anode current collector since sodium has no reaction with aluminum. Therefore, SIB has received considerable attention as a promising alternative to LIB (Dunn et al., 2011; Yang et al., 2011, 2017; Palomares et al., 2013; Pan et al., 2013; Yabuuchi et al., 2014; Han et al., 2015; Kubota and Komaba, 2015; Kundu et al., 2015; Xiang et al., 2015; Hwang et al., 2017; Luo et al., 2017; Nayak et al., 2018; Zhu et al., 2019).

**Figure 1:**
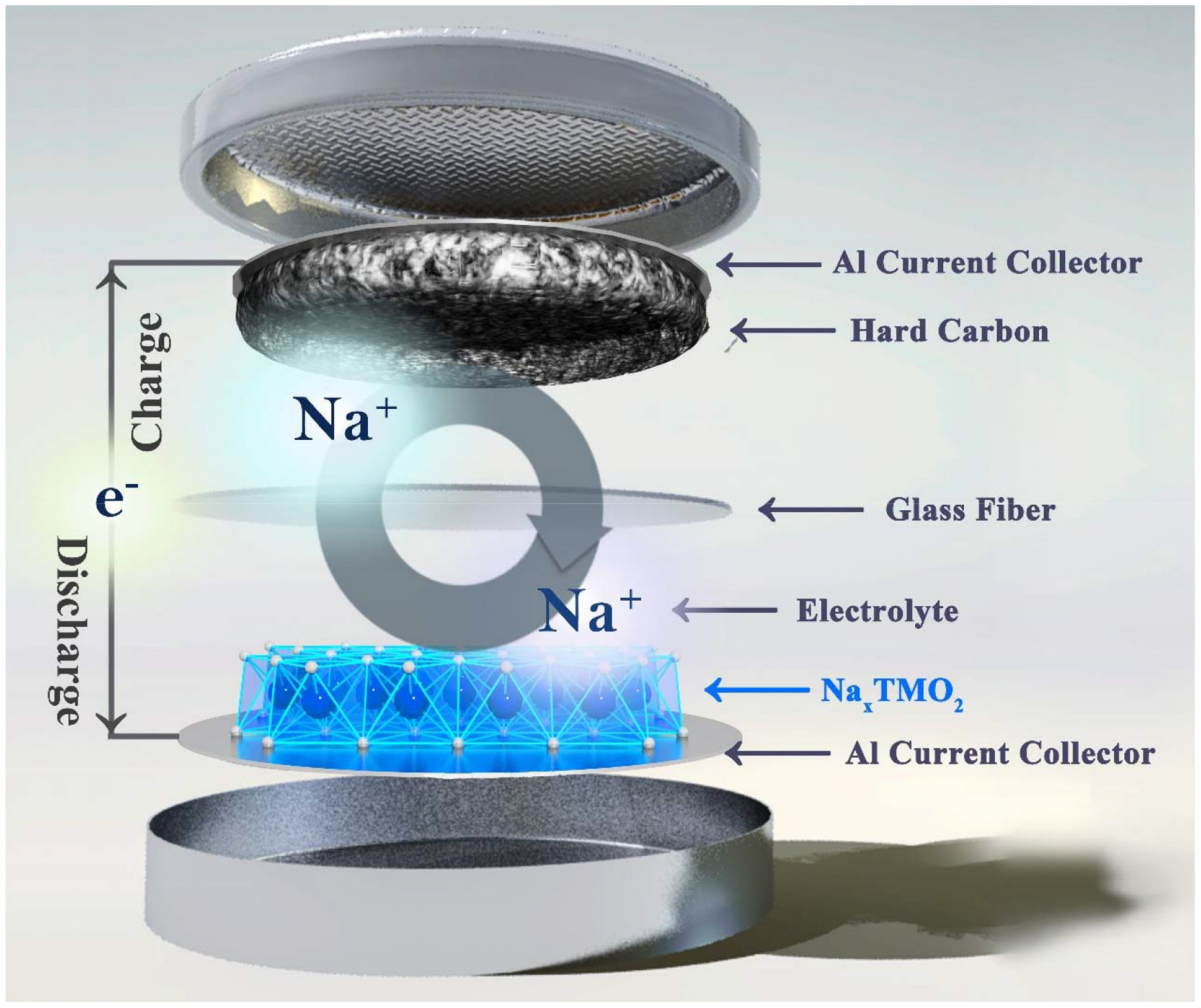
The configuration of sodium-ion battery.

The SIB system consists of five parts: cathode, anode, membrane, electrolyte and current collector, which has the same structure as LIB. Figure 1 shows typical configuration of a SIB coin cell, in which sodium layered transition metal oxide (Na_*x*_TMO_2_) and hard carbon are employed as cathode and anode, respectively. During the charge process, the Na^+^ and e^−^ migrates to hard carbon anode with voltage increasing. During the discharge process, Na^+^ and e^−^ return to Na_*x*_TMO_2_ cathode reversibly with voltage decreasing. The overall reaction can be described as:

(1) $$\begin{array}{r} \text{N}{\text{a}}_{\text{x}}\text{TM}{\text{O}}_{2}+\text{C }↔\text{ TM}{\text{O}}_{2}+\text{N}{\text{a}}_{\text{x}}\text{C} \end{array}$$

Numerous cathode materials such as polyanion compounds (Tripathi et al., 2013; Zhang Y. et al., 2016), layered transition metal (TM) oxides (Roger et al., 2007; Berthelot et al., 2011; Carlier et al., 2011) and Prussian blue or Metal-Organic compounds (Fang et al., 2017; Su et al., 2017; Qian et al., 2018) have been applied as Na^+^ host materials. Layered TM oxides show a high theoretical capacity among these cathode materials (Wang et al., 2018c). In addition, taking the preparation process and cost into consideration, the layered transition metal oxides are the optimal choice for practical application because they can be easily obtained by calcining the precursors in air. As a result, the layered transition metal oxides with general formula Na_*x*_TMO_2_ have attracted more and more attention since the first report by Delmas' group in the 1980s (Delmas et al., 1980, 1981).

Most of researches about Na_*x*_TMO_2_ focused on the improvement of electrochemical properties, such as: (i) eliminating Na^+^ vacancy ordering to improve rate capability; (Wang et al., 2015, 2018a; Kang et al., 2018) (ii) suppressing phase transition or surface coating to achieve long cycling life; (Wang et al., 2016c; Wang P.-F. et al., 2017; You et al., 2017; Sathiya et al., 2018) (iii) exploring oxygen ion redox mechanism to achieve high energy density (Rozier et al., 2015; Kim et al., 2017; Bai et al., 2018; Maitra et al., 2018; Qiao et al., 2018; Rong et al., 2018), and so on. However, most Na_*x*_TMO_2_ materials are hygroscopic and air-instable, which limit their practical applications because huge cost will be spent on materials' storage and transportation (Blesa et al., 1993; Franger et al., 2000; Lu and Dahn, 2001b; Caballero et al., 2002; Monyoncho and Bissessur, 2013; Duffort et al., 2015; Kubota and Komaba, 2015; Boyd et al., 2018; You et al., 2018). So in recent years, the design and synthesis of air-stable Na_*x*_TMO_2_ materials have become a hot topic. In this review, we summarize the recent progress on air-stable Na_*x*_TMO_2_ materials from structure understanding to corresponding solutions, and at the same time we address the remaining problems and challenges for further development.

## Structure of Na*_*x*_*TMO_2_

**Figure 2:**
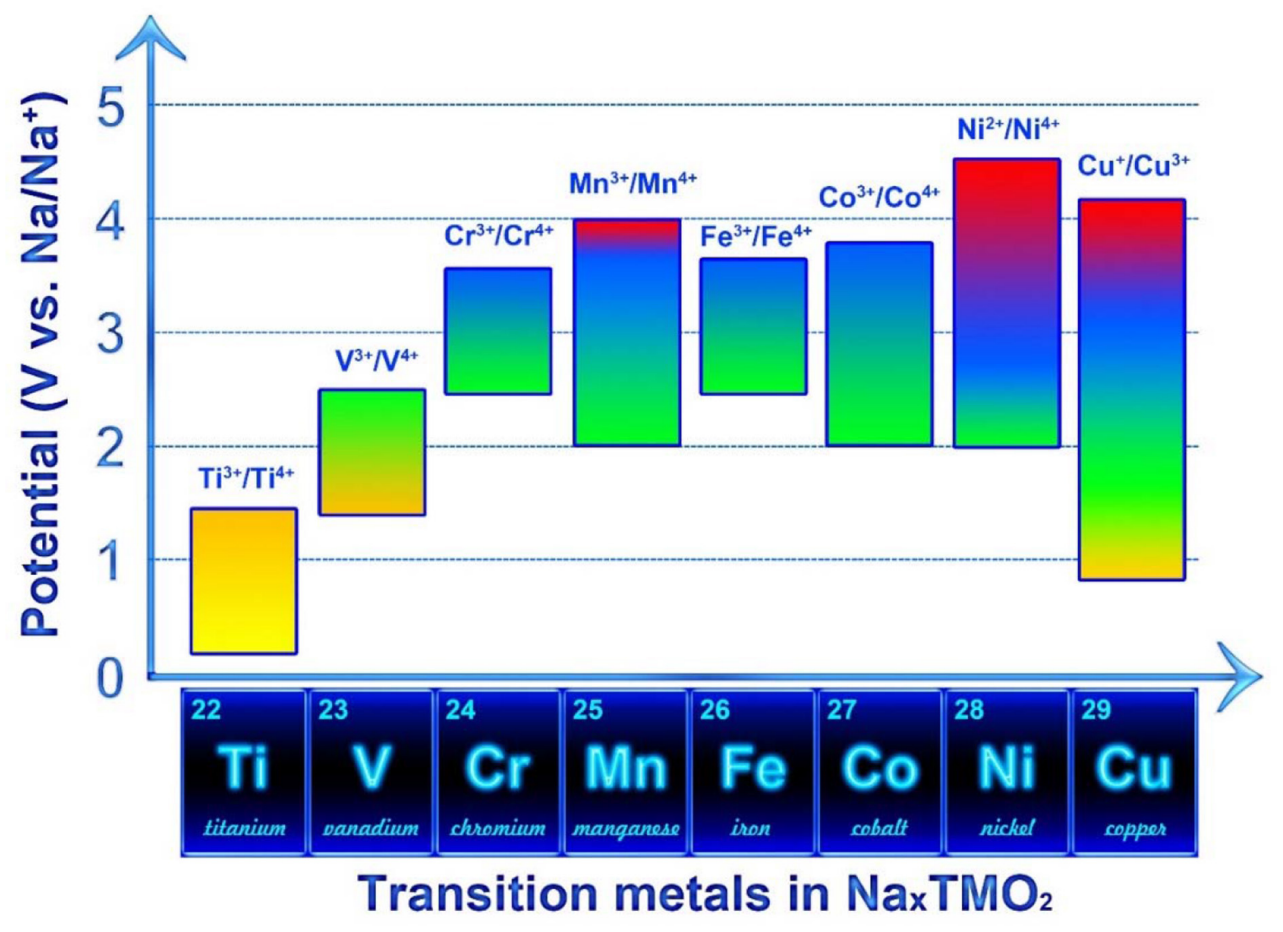
The redox couples in Na_*x*_TMO_2_ compounds with their corresponding potential range.

In Na_*x*_TMO_2_ compounds, TM layers are usually occupied by Ti, (Senguttuvan et al., 2011; Wu D. et al., 2015) V, (Hamani et al., 2011; Guignard et al., 2013; Wang et al., 2018d) Cr, (Braconnier et al., 1982; Yu et al., 2015) Mn, (Ma et al., 2011) Fe, (Blesa et al., 1993; Yabuuchi et al., 2012b) Co, (Berthelot et al., 2011; Rai et al., 2014) Ni, (Vassilaras et al., 2013; Wang et al., 2018b) Cu, (Ono et al., 2014; Jiang et al., 2018; Ono, 2018) a mixture of two (Saadoune et al., 1996; Yabuuchi et al., 2012a; Mortemard de Boisse et al., 2013; Gonzalo et al., 2014; Guo et al., 2014; Kalluri et al., 2014; Zhu et al., 2014, 2016; Chen et al., 2015; Jiang et al., 2015; Kang et al., 2015; Wang et al., 2015, 2016d; Bucher et al., 2016; Kee et al., 2016; Liu et al., 2016; Manikandan et al., 2017; Sabi et al., 2017; Song et al., 2017) or more elements (Lu and Dahn, 2001b; Buchholz et al., 2014; Li et al., 2014; Liu et al., 2015; Li Y. et al., 2015; Li Z.-Y. et al., 2015; Yue et al., 2015; Han et al., 2016a; Kang et al., 2016; Qi et al., 2016; Satyanarayana et al., 2016; Sun et al., 2016; Wang et al., 2016b; Zhang X.-H. et al., 2016; Wang L. et al., 2017; Zheng and Obrovac, 2017) The corresponding redox potential ranges of these TM are presented in Figure 2. Na_*x*_TiO_2_ compound is usually used as anode material due to its low redox potential range. Na_*x*_(Ni_*y*_Mn_1−y_)O_2_ compound has been thoroughly investigated as cathode material because of the relatively high redox potential and theoretical capacity. (Lu and Dahn, 2001a; Fielden and Obrovac, 2015) V, Cr and Co substitution also shows a proper potential range for cathode but it may not suitable for practical application since V, Cr, and Co are expensive and toxic. Although Fe and Cu are almost electrochemical inactive when used as LiTMO_2_ for LIB system, (Ado et al., 1997; Arachi et al., 2012) these two elements are proven to be highly active in Na_*x*_TMO_2_ as Na-ion host. (Yabuuchi et al., 2012a; Ono, 2018) Since Ni and Co resources are mostly consumed by LIB system, the abundant Fe and Cu resources with low price are suitable for Na_*x*_TMO_2_ as sodium storage materials. (Li Y. et al., 2015; Mu et al., 2015) In addition, electrochemical inactive metal ions such as Li^+^, Mg^2+^ and Zn^2+^ could also be introduced into the TM layer for the improvement of electrochemical performance (Xu et al., 2014; Wu X. et al., 2015; Wang et al., 2016c). Table 1 lists the most common metal ions for the construction of TM layers and their corresponding ionic radii with coordination number of six (Shannon, 1976). Cations with similar ionic radius can partially substitute each other to form solid solutions, and hence various Na_*x*_TMO_2_ compounds could be designed by choosing two or more proper metal ions for the TM layer to improve electrochemical performance.ta

**Table 1:** Ionic radius of metal ions.

**Metal ion**	**Coordination number**	**Ionic radius (Å)**
Li^+^	VI	0.76
Mg^2+^	VI	0.72
Al^3+^	VI	0.535
Ti^4+^	VI	0.605
V^5+^	VI	0.54
Cr^3+^	VI	0.615
Mn^4+^	VI	0.53
Fe^3+^	VI	0.55(L); 0.645(H)^*^
Co^3+^	VI	0.545(L); 0.61 (H)
Ni^2+^	VI	0.69
Cu^2+^	VI	0.73
Zn^2+^	VI	0.74

* *L means low spin while H means high spin*.

**Figure 3:**
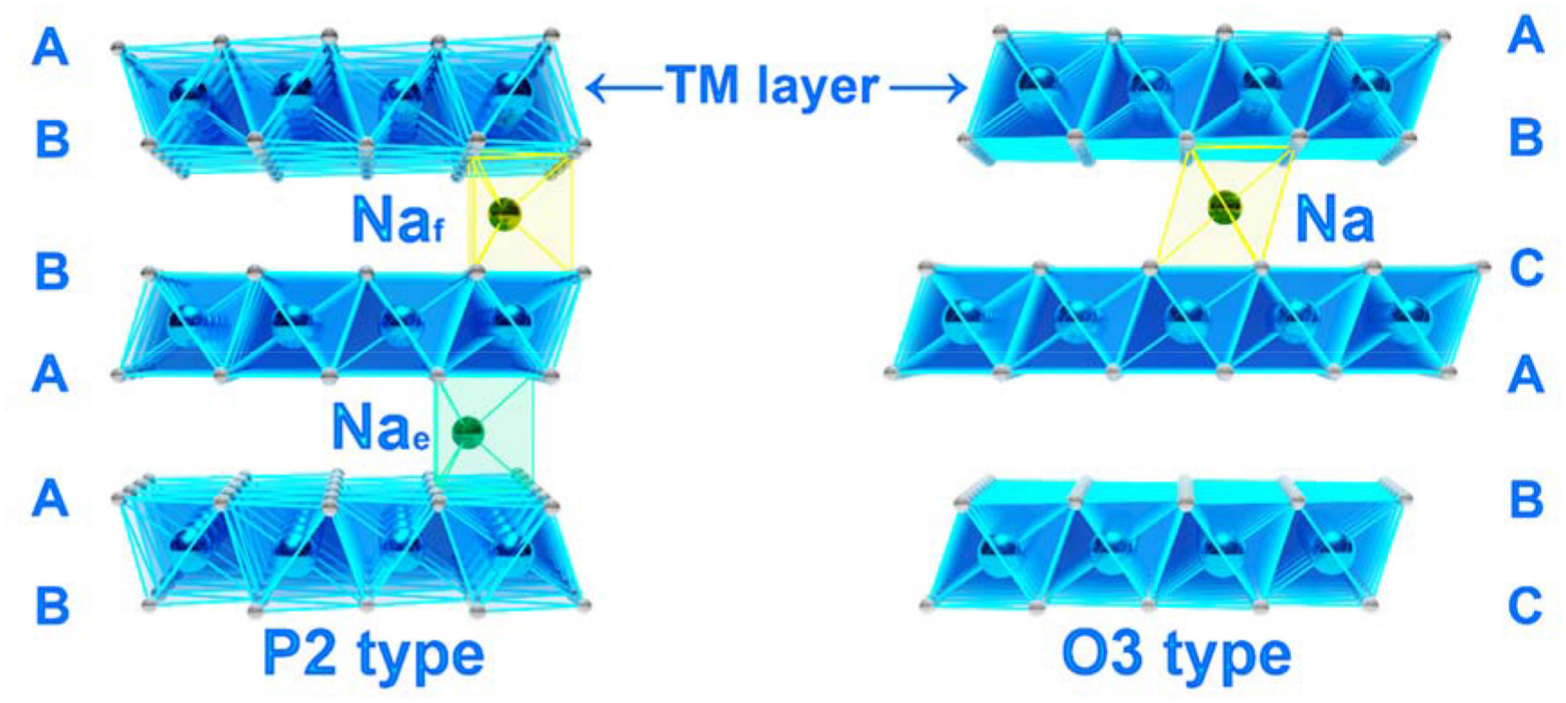
The schematic of the crystal structure for layered sodium TM oxides, left is P2 type and right is O_3_ type.

The crystal structure of Na_*x*_TMO_2_ can be usually classified into two types, P2 and O3 (Figure 3). The symbols of “P” and “O” are from the abbreviation of “prismatic” and “octahedral,” “2” and “3” represents the stacking arrangement per unit of O ions. For P2 type structure (usually *x* = 2/3), Na ions occupy two different prismatic sites, one shares faces between TMO_6_ octahedra called Na_f_ sites and another shares edges between TMO_6_ octahedra called Na_e_ sites. TM ions are surrounded by oxygen frameworks with a stacking mode of ABBA. For O3 structure (usually x = 1), all Na ions share one edge and one face with TMO_6_ octahedra. The oxygen frameworks are arranged in ABCABC pattern (Delmas et al., 1980, 1981; Shu and Chou, 2008; Morris et al., 2009; Toumar et al., 2015; Zheng C. et al., 2017).

### Influence of Air on Na*_*x*_*TMO_2_

**Figure 4:**
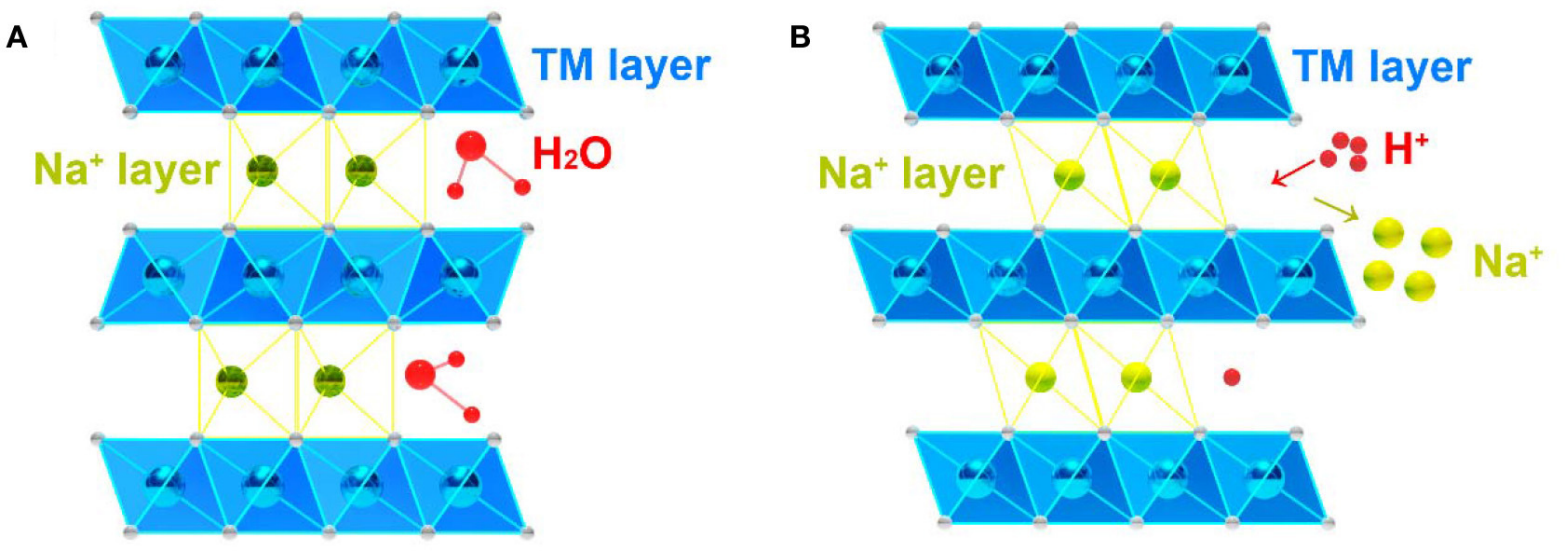
**(A)** Insertion of water molecules in Na^+^ layer, **(B)** Ion exchange between H^+^ and Na^+^ in Na^+^ layer.

So far, many researches have proven that the water and CO_2_ molecules from air can react with Na_*x*_TMO_2_, bringing negative influence on its morphology, crystal structure and electrochemical performance. The water molecules are easy to react with air-instable Na_*x*_TMO_2_ by inserting into the Na layer (Le Goff et al., 1993; Paulsen and Dahn, 1999; Franger et al., 2000; Lu and Dahn, 2001b; Caballero et al., 2002; Duffort et al., 2015; Boyd et al., 2018) or exchanging Na^+^ with H^+^, (Blesa et al., 1993; Monyoncho and Bissessur, 2013; Kubota and Komaba, 2015; Han et al., 2016b; Yao et al., 2017) leading to the expansion of interlayer spacing and the formation of impure phase (Figure 4). While CO_2_ can transform to CO${}_{3}^{2-}$ on the surface of Na_*x*_TMO_2_ (Duffort et al., 2015; You et al., 2018). These air exposed Na_*x*_TMO_2_ usually show serious capacity decay and large polarization because of: (i) the side reaction between water and electrolyte (Kawamura et al., 2006; Lux et al., 2012; Han et al., 2016b); (ii) the active-materials' surface dissolution triggered by the acid attack of proton, which is released by water molecules (Blyr et al., 1998; Thackeray et al., 1998; Benedek and van de Walle, 2008); (iii) capacity and electronic conductivity decrease caused by inactive Na_2_CO_3_ layer (Duffort et al., 2015; You et al., 2018). Therefore, the air-instable Na_*x*_TMO_2_ cannot maintain its original crystal structure and electrochemical property under moisture atmosphere condition.

### Reaction Mechanisms of Water on Na*_*x*_*TMO_2_

**Figure 5:**
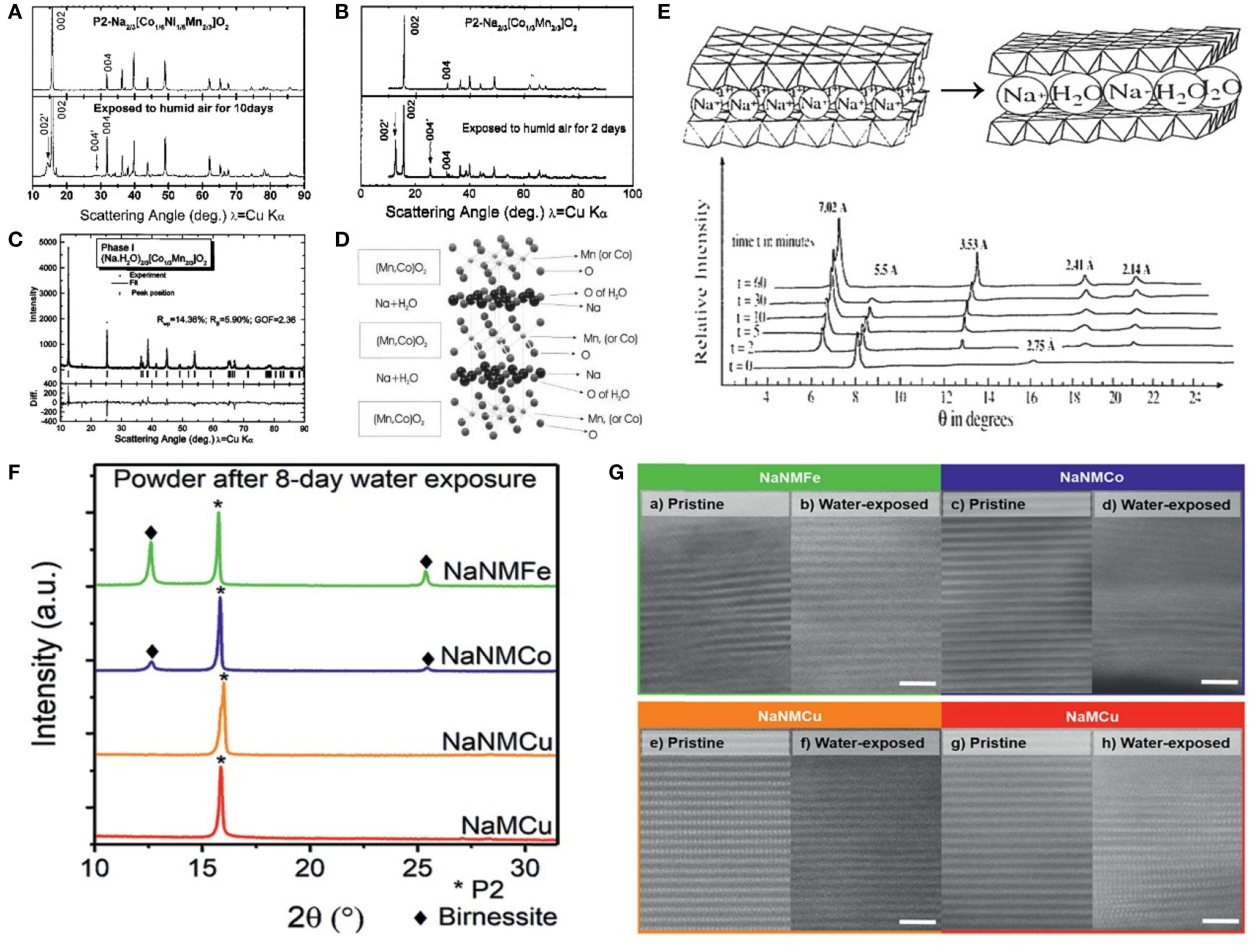
**(A)** XRD patterns of pristine and air-exposed Na_2/3_Co_1/6_Ni_1/6_Mn_2/3_O_2_ samples, **(B)** XRD patterns of pristine and air-exposed Na_2/3_Co_1/3_Mn_2/3_O_2_ samples, **(C)** Rietveld refinement of (Na·H_2_O)_2/3_Co_1/3_Mn_2/3_O_2_, **(D)** Crystal structure of (Na·H_2_O)_2/3_Co_1/3_Mn_2/3_O_2_. Reproduced with permission (Lu and Dahn, 2001b). Copyright 2001, American Chemical Society. **(E)** Water molecules insert into the Na layer of Na_0.7_MnO_2_ and the change of XRD patterns with the increasing time of water soaking. Reproduced with permission (Franger et al., 2000). Copyright 2000, The Electrochemical Society. **(F)** XRD patterns of P2-Na_0.62_Ni_0.22_Mn_0.66_Fe_0.1_O_2_ (NaNMFe), P2-Na_0.61_Ni_0.22_Mn_0.66_Co_0.1_O_2_ (NaNMCo), P2-Na_0.64_Ni_0.22_Mn_0.66_Cu_0.11_O_2_ (NaNMCu) and P2-Na_0.64_Mn_0.62_Cu_0.31_O_2_ (NaMCu) samples after water soaking, **(G)** STEM images of pristine and water soaked NaNMFe, NaNMCo, NaNMCu and NaMCu samples. Reproduced with permission (Boyd et al., 2018). Copyright 2018, Royal Society of Chemistry.

Water molecules can insert into Na layer to form a Na_*x*_TMO_2_·yH_2_O hydrate phase, which usually occurs in P2 type structure (Le Goff et al., 1993; Franger et al., 2000; Lu and Dahn, 2001b; Caballero et al., 2002; Duffort et al., 2015; Boyd et al., 2018). In 2001, Lu et al. (Lu and Dahn, 2001b) studied the water insertion reaction mechanism for the first time in P2-Na_2/3_Co_*x*_Ni_1/3−x_Mn_2/3_O_2_ compound (*x* = 1/6 or 1/3). Compared with the XRD patterns of pristine Na_2/3_Co_*x*_Ni_1/3−x_Mn_2/3_O_2_, two new peaks around 14° and 28° were observed after exposing Na_2/3_Co_*x*_Ni_1/3−x_Mn_2/3_O_2_ samples in humid air environment (Figures 5A,B). These two peaks were assigned as hydrate phase due to the insertion of water molecules in Na layers. Rietveld refinement of hydrate Na_2/3_Co_1/3_Mn_2/3_O_2_·yH_2_O indicated that the ratio of water/Na is close to 1:1 and the oxygen atoms from water was in the 2c site of the crystal structure (Figures 5C,D). Franger et al. (Franger et al., 2000) investigated the influence of water soaking on α-Na_0.7_MnO_2_. With the increasing of water soaking time, the two peaks around 8° and 16° were vanished and four new peaks around 6.5°, 13°, 19° and 21° appeared gradually. The α-Na_0.7_MnO_2_ was totally transformed to Na_0.45_MnO_2_·0.6H_2_O after 60 min of water soaking treatment (Figure 5E). In 2018, Boyd et al. compared the air-stability of P2-Na_0.62_Ni_0.22_Mn_0.66_Fe_0.1_O_2_ (NaNMFe), P2-Na_0.61_Ni_0.22_Mn_0.66_Co_0.1_O_2_ (NaNMCo), P2-Na_0.64_Ni_0.22_Mn_0.66_Cu_0.11_O_2_ (NaNMCu) and P2-Na_0.64_Mn_0.62_Cu_0.31_O_2_ (NaMCu) samples. After air-exposure treatment of these four samples for 8 days, the XRD patterns of NaNMCu and NaMCu samples remained unchanged, while two new peaks around 12.5° and 25° appeared in the patterns of NaNMFe and NaNMCo samples, indicating that water can insert in the interlayer spacing of NaNMFe and NaNMCo samples (Figure 5F). From the STEM images of these four samples before and after air-exposure, an obvious extension in interlayer spacing could be seen after air-exposure, proving the insertion of water molecules in the interlayer spacing (Figure 5G). Although water molecules can insert into the interlayer spacing of P2-Na_*x*_TMO_2_ to form a P2-Na_*x*_TMO_2_·yH_2_O hydrate phase, Na_*x*_TMO_2_ phase can be regenerated by heat treatment at 200 °C to remove the water molecules (Lu and Dahn, 2001b).

For most O3-type NaTMO_2_, water molecules can release H^+^ to exchange the Na^+^, (Blesa et al., 1993; Monyoncho and Bissessur, 2013; Kubota and Komaba, 2015; Han et al., 2016b; Yao et al., 2017) which could be regarded as hydrolysis reaction:

(2) $$\begin{array}{r} \text{NaTM}{\text{O}}_{2}+\text{x}{\text{H}}_{2}\text{O}\to \text{N}{\text{a}}_{1-\text{x}}{\text{H}}_{\text{x}}\text{TM}{\text{O}}_{2}+\text{NaOH} \end{array}$$

Specially, if the TM layers contain a certain amount of Ni^2+^ ions, NiO would be emerged during the air exposure treatment:

(3) $$\begin{array}{l} {\text{NaNi}}_{\text{y}}{\text{TM}}_{1-\text{y}}{\text{O}}_{2}+{\text{xH}}_{2}\text{O}\to {\text{Na}}_{1-\text{x}}{\text{H}}_{\text{x}}\text{Niy}-{\text{zTM}}_{1-y}{\text{O}}_{2-\text{z}}\\ \;+\text{xNaOH}+\text{zNiO} \end{array}$$

**Figure 6:**
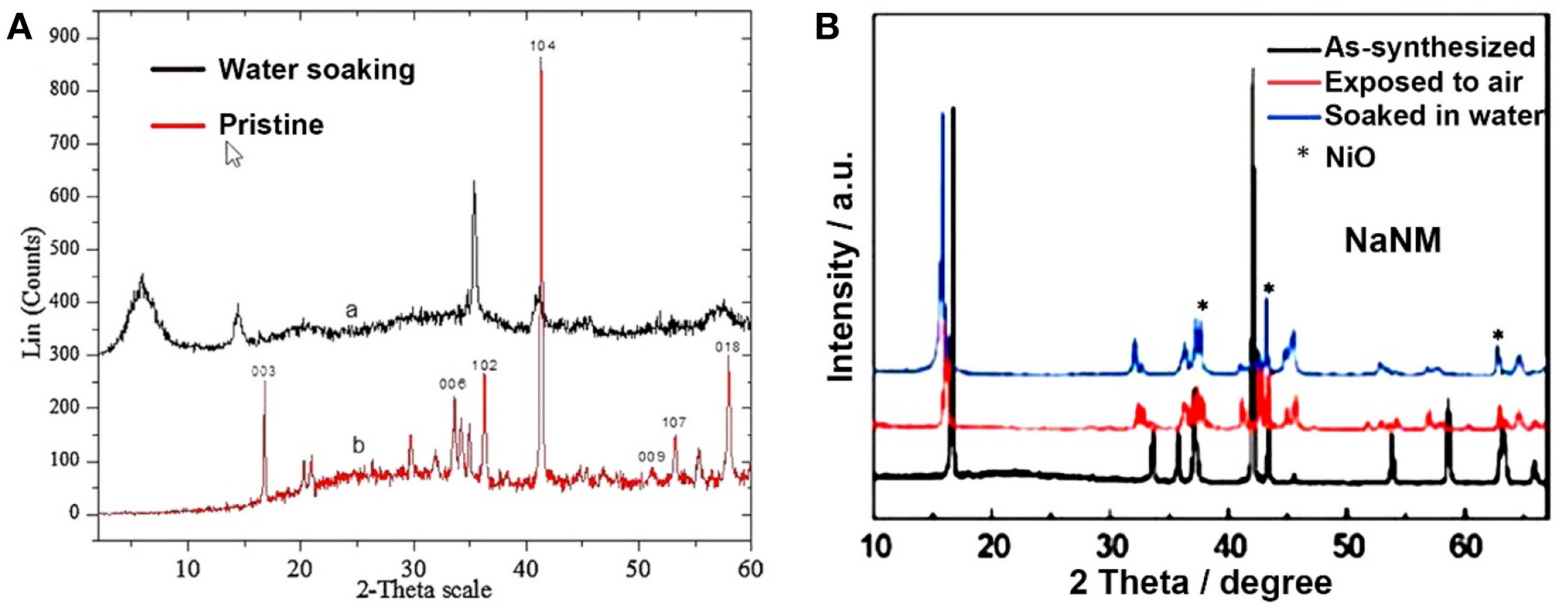
**(A)** XRD patterns of pristine and water soaked NaFeO_2_ samples. Reproduced with permission (Monyoncho and Bissessur, 2013). Copyright 2013, Elsevier. **(B)** XRD patterns of pristine, air exposed and water soaked NaNi_0.5_Mn_0.5_O_2_ samples. Reproduced with permission (Yao et al., 2017). Copyright 2017, American Chemical Society.

This hydrolysis phenomenon has been confirmed in NaNi_0.5_Mn_0.5_O_2_, NaNi_0.7_Mn_0.15_Co_0.15_O_2_ and NaFeO_2_ compounds (Blesa et al., 1993; Monyoncho and Bissessur, 2013; Kubota and Komaba, 2015; You et al., 2018). A simple way to verify this hydrolysis reaction is to analyze the change of pH value after soaking NaTMO_2_ in deionized water due to the release of NaOH (Blesa et al., 1993). In 2013, Monyoncho and Bissessur reported that the pH of aqueous solution was higher than 12 after mixing O3-NaFeO_2_ sample with deionized water (Monyoncho and Bissessur, 2013). In addition, compared with the XRD patterns of pristine NaFeO_2_ sample, the 003 peak of hydrolysis Na_1−x_H_x_FeO_2_ sample became broader and shifted to low angle (Figure 6A), indicating the formation of a disordered crystal structure. Wang et al. investigated the hydrolysis reaction of O3-NaNi_0.5_Mn_0.5_O_2_ sample by testing the temperature after water soaking because this reaction can release heats (Yao et al., 2017). After NaNi_0.5_Mn_0.5_O_2_ was added into water, the temperature of the water was increased from 24.4 to 30.8°C. In contrast to the XRD pattern of as-synthesized sample, the 003 and 006 peaks of water soaked NaNi_0.5_Mn_0.5_O_2_ shifted to low angle with the generation of NiO impurity phase (Figure 6B). Importantly, unlike P2 type, the hydrolysis reaction between O3 type NaTMO_2_ and water is irreversible.

### Reaction Mechanisms of CO_2_ on Na_x_TMO_2_

As mentioned above, NaOH is generated on the surface of O3 type Na_*x*_TMO_2_ during the air exposure process, then CO_2_ can further react with NaOH to form Na_2_CO_3_ (Sathiya et al., 2012; Monyoncho and Bissessur, 2013; You et al., 2018). This reaction can be described as:

(4) $$\begin{array}{r} \text{NaTM}{\text{O}}_{2}+\text{x}{\text{H}}_{2}\text{O}+\text{C}{\text{O}}_{2}\to \text{N}{\text{a}}_{1-\text{x}}{\text{H}}_{\text{x}}\text{TM}{\text{O}}_{2}+\text{N}{\text{a}}_{2}\text{C}{\text{O}}_{3} \end{array}$$

**Figure 7:**
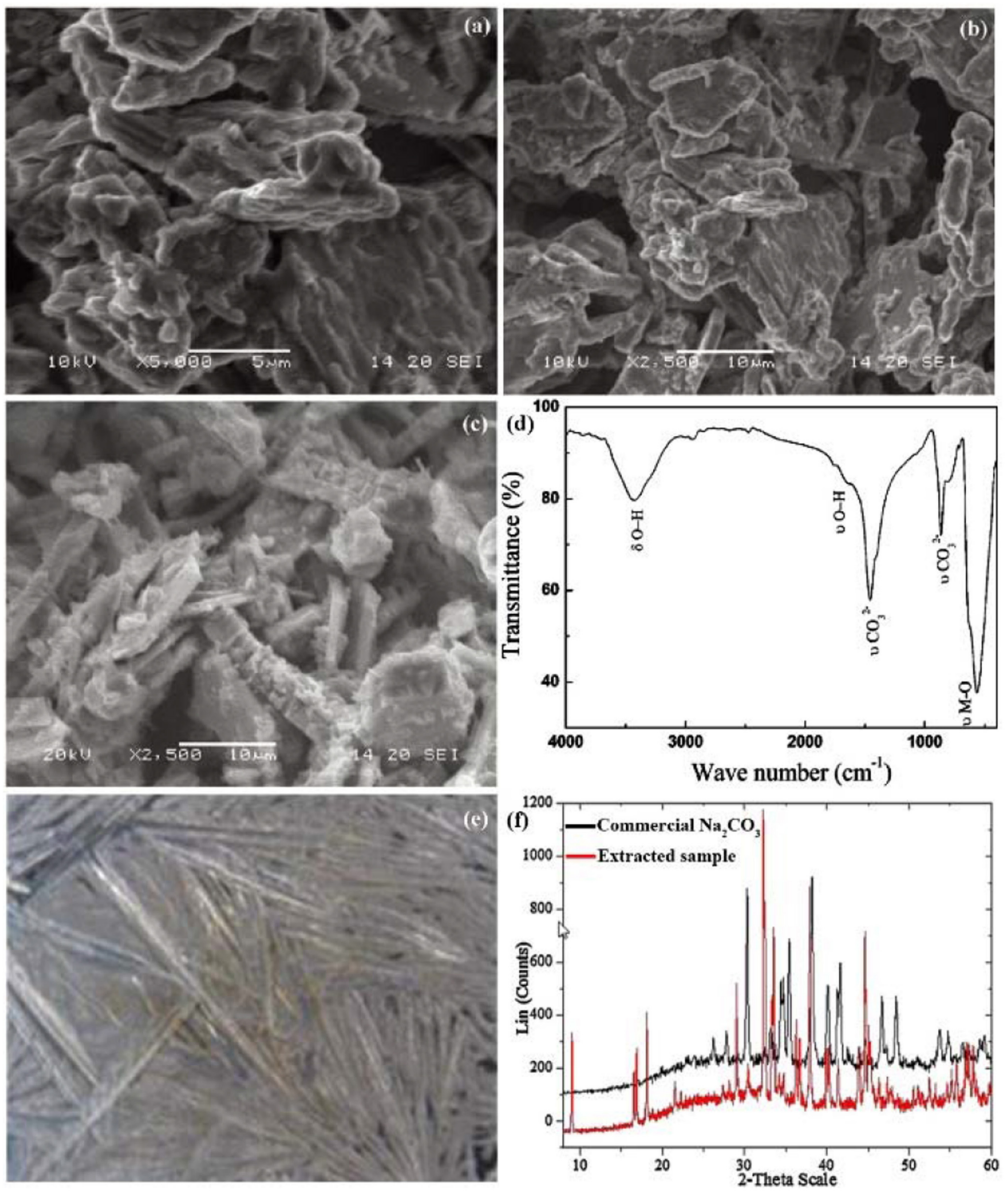
SEM images of **(a)** pristine NaNi_1/3_Mn_1/3_Co_1/3_O_2_, **(b)** NaNi_1/3_Mn_1/3_Co_1/3_O_2_ after 15 days air-exposure, **(c)** NaNi_1/3_Mn_1/3_Co_1/3_O_2_ after 30 days air-exposure. **(d)** Infrared spectrum of NaNi_1/3_Mn_1/3_Co_1/3_O_2_ after 30 days air-exposure. Reproduced with permission (Sathiya et al., 2012). Copyright 2012, American Chemical Society. **(e)** SEM image of extracted sample, the sample is prepared by drying the aqueous solution of water-NaFeO_2_ mixture, **(f)** XRD patterns of the extracted sample and commercial Na_2_CO_3_. Reproduced with permission (Monyoncho and Bissessur, 2013). Copyright 2013, Elsevier.

Sathiya et al. (2012) proved the formation of Na_2_CO_3_ on the surface of NaNi_1/3_Mn_1/3_Co_1/3_O_2_ particles. Compared to the pristine sample (Figure 7a), the surface showed no obvious change after 15 days air-exposure (Figure 7b) but became quite rough after 30 days air-exposure (Figure 7c). IR spectrum revealed the bands of CO${}_{3}^{2-}$ at 1,450 and 863 cm^−1^, suggesting the existence of sodium carbonates on NaNi_1/3_Mn_1/3_Co_1/3_O_2_ particles' surface (Figure 7d). Monyoncho and Bissessur (2013) extracted the aqueous solution from the mixture of NaFeO_2_ and water (Figure 7e). The XRD pattern of extracted sample matched very well to commercial Na_2_CO_3_, proving the reaction of CO_2_ and NaFeO_2_ compounds (Figure 7f).

**Figure 8:**
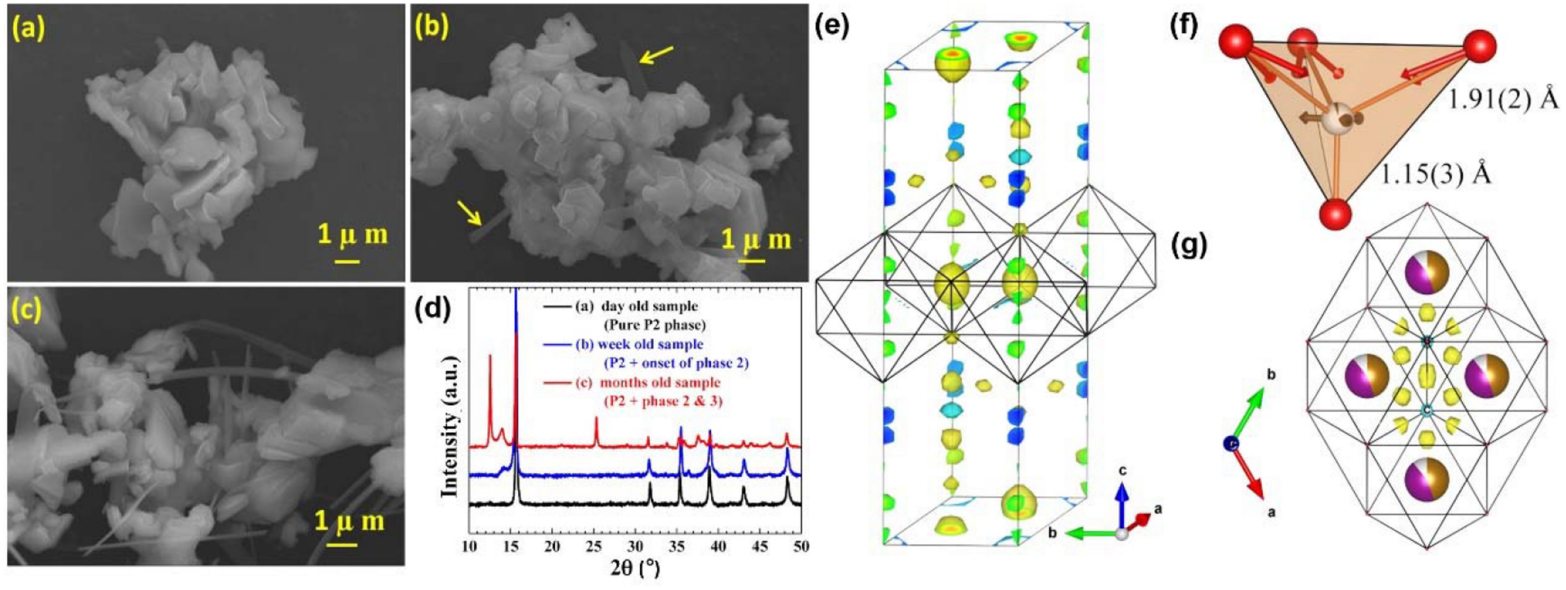
**(a)** SEM images of Na_0.67_Mn_0.5_Fe_0.5_O_2_ after 1 day air-exposure, **(b)** Na_0.67_Mn_0.5_Fe_0.5_O_2_ after a couple of weeks' air-exposure, **(c)** Na_0.67_Mn_0.5_Fe_0.5_O_2_ after a couple of months' air-exposure, **(d)** XRD patterns of Na_0.67_Mn_0.5_Fe_0.5_O_2_ samples under different air-exposure time, **(e)** Nuclear Fourier difference map of Na_0.67_Mn_0.5_Fe_0.5_O_2_, **(f)** The local environment of CO, **(g)** The nuclear density around the carbon element position. Reproduced with permission (Duffort et al., 2015). Copyright 2015, American Chemical Society.

**Figure 9:**
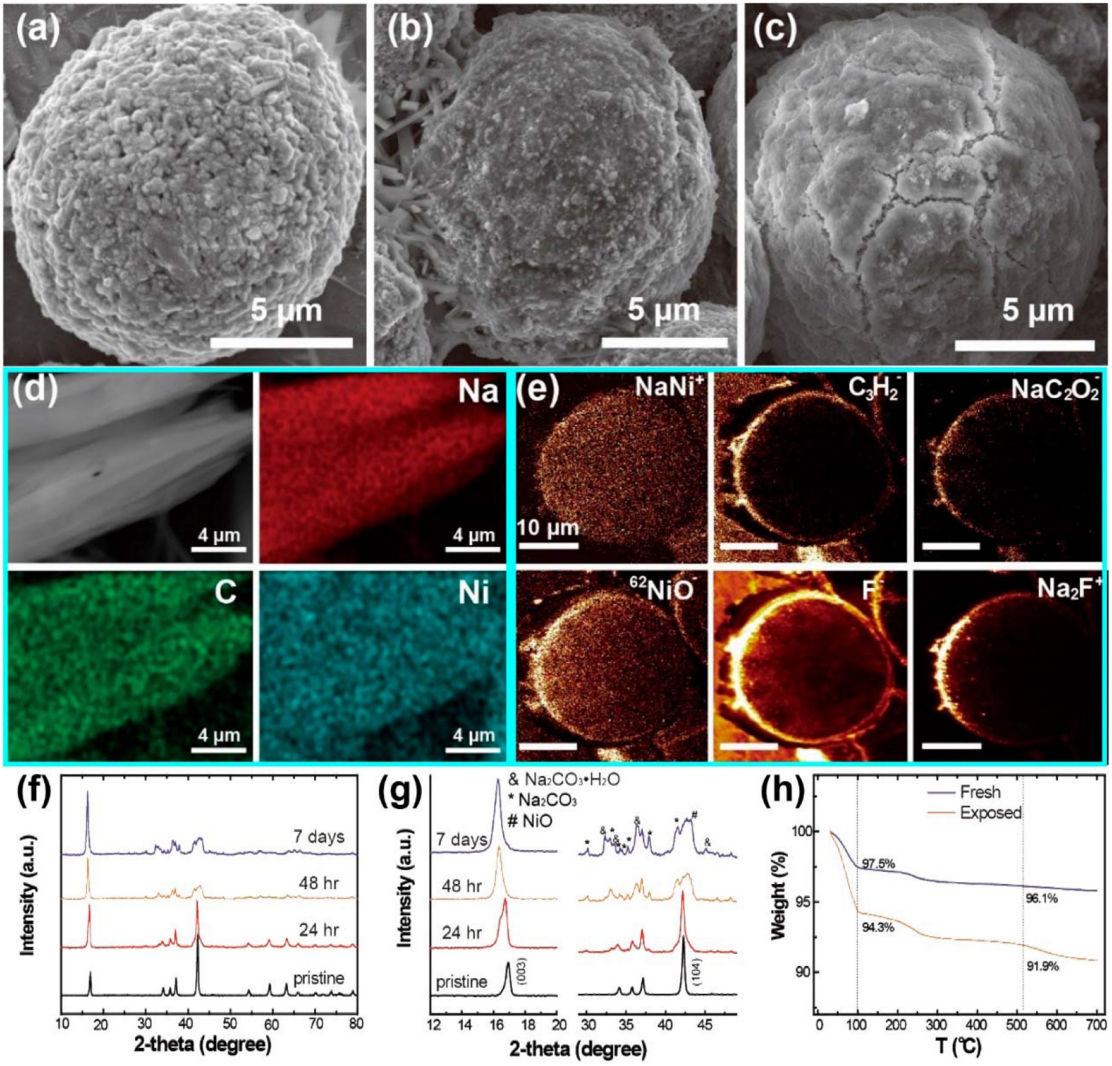
SEM images of **(a)** pristine NaNi_0.7_Mn_0.15_Co_0.15_O_2_, **(b)** NaNi_0.7_Mn_0.15_Co_0.15_O_2_ after 24 h air-exposure, **(c)** NaNi_0.7_Mn_0.15_Co_0.15_O_2_ after 7 days air-exposure, **(d)** dendrite-like impurity in part b with the corresponding elemental mappings of Na, C and Ni, **(e)** TOF-SIMS chemical mapping of NaNi_0.7_Mn_0.15_Co_0.15_O_2_ after 24 h exposure, **(f)** XRD patterns of NaNi_0.7_Mn_0.15_Co_0.15_O_2_ samples under different air exposed time, **(g)** the enlarged peaks from part f, **(h)** TGA profiles of pristine NaNi_0.7_Mn_0.15_Co_0.15_O_2_ and 7 days exposed NaNi_0.7_Mn_0.15_Co_0.15_O_2_ samples. Reproduced with permission (You et al., 2018). Copyright 2018, American Chemical Society.

After the formation of Na_2_CO_3_ on the surface, the CO${}_{3}^{2-}$ can even be inserted into the TM layer, forming a “CO_4_” tetrahedron. Duffort et al. elucidated the mechanism of CO${}_{3}^{2-}$ insertion in Na_0.67_Mn_0.5_Fe_0.5_O_2_ crystal (Duffort et al., 2015). With increasing the time of air exposure, ribbon-like particles start to appear and grow longer gradually (Figures 8a–c). In addition, the corresponding XRD patterns of Na_0.67_Mn_0.5_Fe_0.5_O_2_ are also changed. New peak is observed around 13° after a month air exposure (Figure 8d), indicating the formation of hydrate phase (phase 3) and sodium-depleted P2 phase (phase 2). In Fourier difference map, the existence of large residual nuclear density is caused by the carbonate ions (Figure 8e) because the insertion of CO${}_{3}^{2-}$ leading to the changing of the nuclear density distribution (Figure 9g). In the TM layer, the CO${}_{3}^{2-}$ is combined with one C-O bond to form a CO_4_ tetrahedron structure (Figure 8f).

Except for Na_2_CO_3_, other surface components are also observed. You et al. studied the surface reaction between NaNi_0.7_Mn_0.15_Co_0.15_O_2_ and air systematically by using time-of-flight secondary ion mass spectroscopy (TOF-SIMS) (You et al., 2018). The pristine sample shows a microsphere morphology, which is consisted of nanosized particles (Figure 9a). After 24 h air exposure treatment, the surface of this microsphere becomes smooth with the absence of nano-particles (Figure 9b). Finally, a thick layer of impurities is formed on the surface after 7 days air exposure (Figure 9c). Elements of Na, Ni, and C are distributed uniformly on the impurity surface (Figure 9d). The existence of NaNi^+^, NiO, NaC_2_O${}_{2}^{-}$, C_3_H${}_{2}^{-}$, Na_2_F^+^, and F^−^ composition are confirmed by TOF-SIMS, indicating the surface degradation of NaNi_0.7_Mn_0.15_Co_0.15_O_2_ as well as the reaction between sodium carbonates and PVDF (Figure 9e). The 003 peak shifts to lower angles gradually because of the migration of Na^+^ to the surface while the 104 peak becomes weak and vanishes after 48 h air exposure (Figure 9f). According to the XRD patterns in Figure 9g, impurities' peaks such as NiO and Na_2_CO_3_ are observed, indicating the surface reaction when NaNi_0.7_Mn_0.15_Co_0.15_O_2_ contacts to CO_2_ and H_2_O. Since the CO_2_ and H_2_O are absorbed and reacted with NaNi_0.7_Mn_0.15_Co_0.15_O_2_, the air-exposed sample loses more weight than the fresh sample (Figure 9h). All the results above can prove hat NaNi_0.7_Mn_0.15_Co_0.15_O_2_ can react with the water and carbon dioxide in the air and the impurities of NaNi^+^, NiO, NaC_2_O${}_{2}^{-}$, C_3_H${}_{2}^{-}$, Na_2_F^+^ and F^−^ are generated on the surface.

## Air-Stable Na*_*x*_*TMO_2_ Compounds

As mentioned above, NaTMO_2_ compounds can react with water and CO_2_ in air, which lead to: (i) the formation of impure phase on the surface; (ii) the insertion of H_2_O and CO${}_{3}^{2-}$ into interlayer spacing and TM layers, respectively. On one side, the formed NaOH and Na_2_CO_3_ impure phase are electrochemical inactive and have low conductivity hence the rate capability of Na_*x*_TMO_2_ suffer serious decrease. On the other side, the water molecules can bring side reaction with electrolyte while the insertion of CO${}_{3}^{2-}$ affects the valence state of TM ions, leading to severe capacity decay. Therefore, more and more researchers are focusing on strategies to address this air-instable problems.

One strategy is to prevent the materials from contacting moisture. During the materials preparation process, once the high-temperature treatment is done, the Na_*x*_TMO_2_ products are transferred to drying room (Wang et al., 2016a) or argon-filled glove box (Yabuuchi et al., 2012a; Vassilaras et al., 2014) immediately for the cooling process and subsequent cell assembling. However, this strategy may not be suitable for the large-scale application because huge cost will rise for materials' storage. Another strategy is to design air-stable Na_*x*_TMO_2_ material. Recently, several P2 and O3 type Na_*x*_TMO_2_ materials with high stability against moisture have been reported (Lu and Dahn, 2001b; Li Y. et al., 2015; Mu et al., 2015; Guo et al., 2017; Yao et al., 2017; Zheng L. et al., 2017; Chen et al., 2018; Deng et al., 2018). Under the treatment of air exposure and water soaking, these air-stable cathodes can maintain their original crystal structure and electrochemical performance. In this part, several effective strategies for air-stable Na_*x*_TMO_2_ designing are summarized.

### Constructing TM Cationic Ordering Arrangement

**Figure 10:**
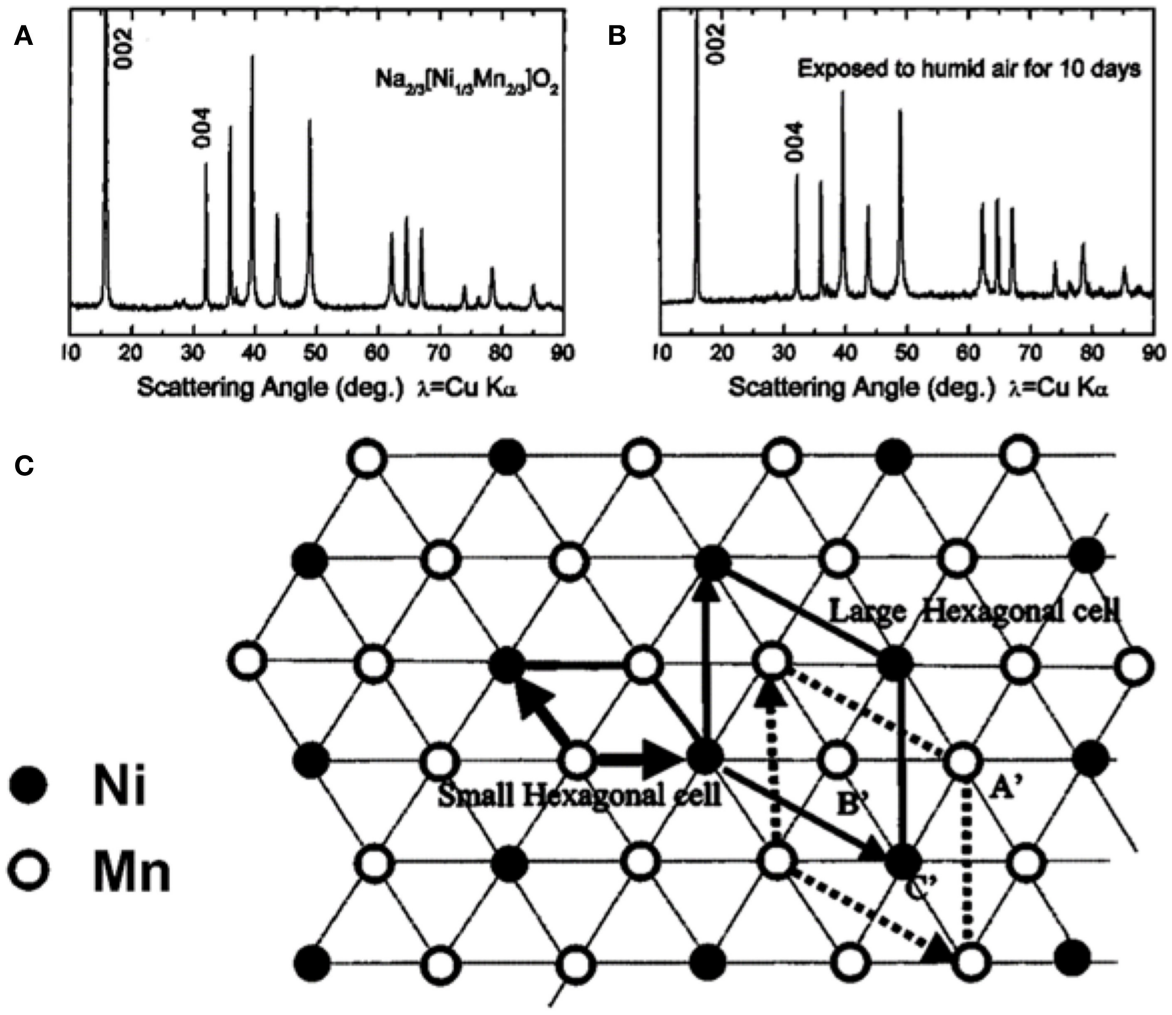
**(A)** XRD pattern of pristine Na_2/3_Ni_1/3_Mn_2/3_O_2_, **(B)** XRD pattern of Na_2/3_Ni_1/3_Mn_2/3_O_2_ after 10 days air-exposure, **(C)** the Ni/Mn cationic ordering arrangement. Reproduced with permission (Lu and Dahn, 2001b). Copyright 2001, American Chemical Society.

Lu et al. first reported an air-stable P2-Na_2/3_Ni_1/3_Mn_2/3_O_2_ with high stability under moisture condition (Lu and Dahn, 2001b). For the P2-Na_2/3_Ni_1/3_Mn_2/3_O_2_ sample, after undergoing a 10 days air-exposure treatment, no peaks shift or new peaks formation were observed in the XRD pattern, indicating that water could not be inserted into the interlayer spacing (Figures 10A,B). According to neutron diffraction analysis, the Ni^2+^ and Mn^4+^ ions formed an honeycomb structure with an √3a × √3a ordering arrangement in the TM layers (Figure 10C) (Lu et al., 2000). This Ni/Mn ordering arrangement in TM layers was supposed to induce a strong interlayer interaction to prevent the water insertion. When this ordering arrangement was suppressed by the substitution of Co or Fe for Ni, water molecules could be inserted into the interlayer spacing (Figures 5A,B,F). However, this “interlayer interaction” between adjacent ordering TM layer has not been confirmed yet.

### Coating Protective Layer

**Figure 11:**
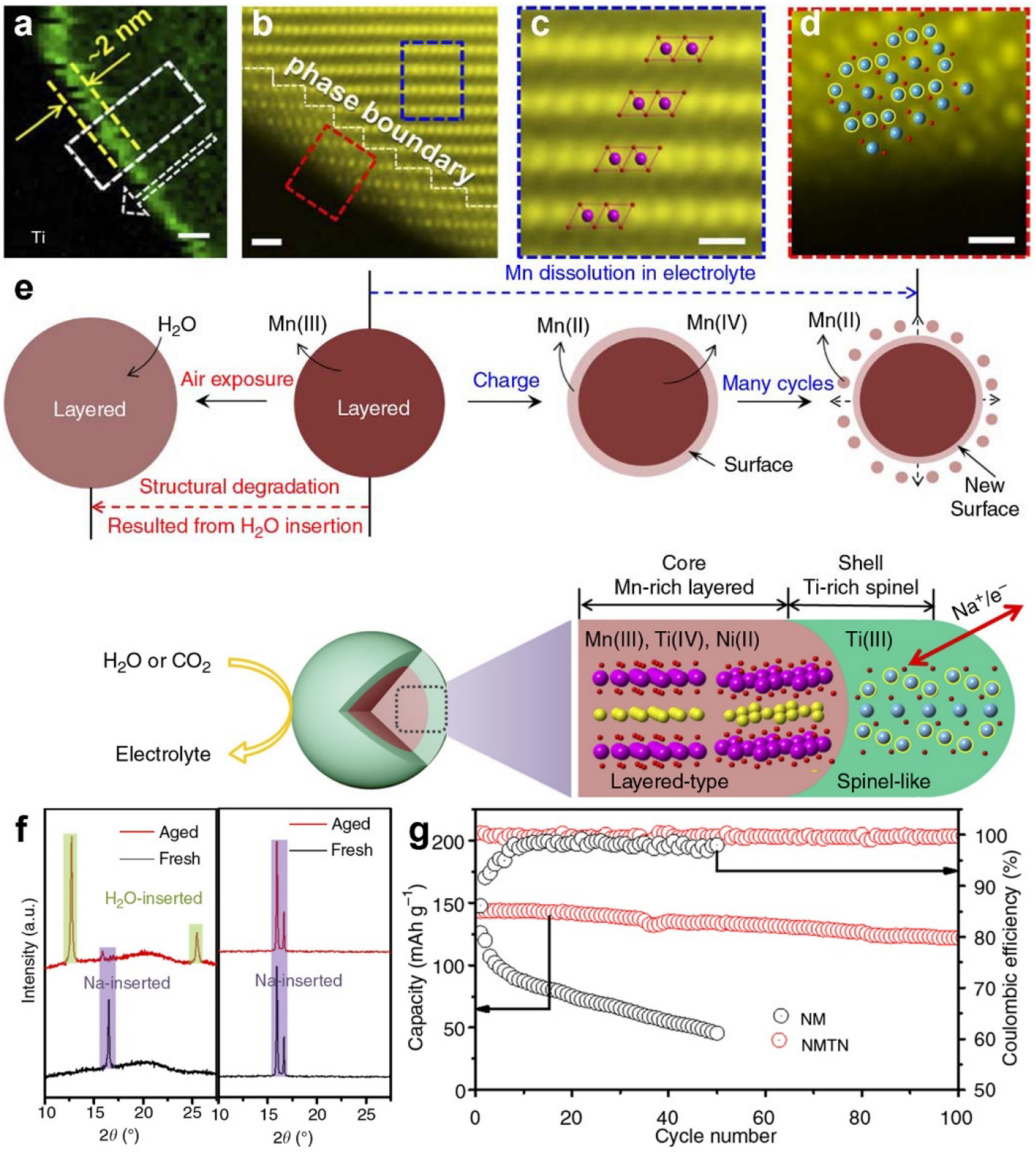
**(a)** EELS chemical mapping of Ti element in NMTN sample, **(b)** STEM-HADDF image of NMTN sample, **(c)** Enlarged image extracted from the blue rectangle of part b, **(d)** Enlarged image extracted from the red rectangle of part b, **(e)** vulnerable mechanism of naked samples and protective mechanism of NMTN sample under moisture condition. **(f)** XRD patterns of naked sample (left) and NMTN sample (right) before/after air-exposure, **(g)** cycling performance of naked sample and NMTN sample. Reproduced with permission (Guo et al., 2017). Copyright 2017, Nature Publishing Group.

Coating a protective layer on the surface of Na_*x*_TMO_2_ is an effective method to prevent the Na_*x*_TMO_2_ from air contacting. The most common way is to coat high-voltage metal oxides with high stability against moisture. In 2017, Zhou and co-workers (Guo et al., 2017). designed an efficient spinel-like titanium (III) oxides protective interface to improve the structure/electrochemical stability of NaMnTi_0.1_Ni_0.1_O_2_. The sample surface was covered by a high Ti concentration layer with thickness of 2 nm, as shown in the electron energy-loss spectroscopy image (Figure 11a). Two distinct phases could be observed from the high-angle annular dark field scanning transmission electron microscopy (HAADF-STEM) image (Figure 11b). The bulk phase was a typical layered structure (Figure 11c) while the surface phase was spinel-like structure (Figures 11d,e). After exposing the naked bulk phase in humid air, two new peaks appeared around 12° and 25° (Figure 11f), indicating the insertion of water molecules. Compared to the naked bulk phase, the XRD pattern of NaMnTi_0.1_Ni_0.1_O_2_ sample showed no peak change since the spinel-like titanium (III) oxides interface can act as shield to protected the bulk phase from water attacking and the electrochemical performance of bulk phase can be maintained. In half cell system, the naked bulk phase showed a dramatic decrease after 50 cycles whereas NMTN sample only showed a slight decay after 100 cycles (Figure 11g).

You et al. (2018) coated the surface of NaNi_0.7_Mn_0.15_Co_0.15_O_2_ with a ZrO_2_ protective layer. This protective layer notably maintains the rate capability of NaNi_0.7_Mn_0.15_Co_0.15_O_2_ against moisture atmosphere. After 7 days air exposure, the ZrO_2_@NaNi_0.7_Mn_0.15_Co_0.15_O_2_ sample still delivers a capacity of 96 mAh/g while NaNi_0.7_Mn_0.15_Co_0.15_O_2_ shows abnormal charge profile. The surface charge-transfer kinetics are also improved by this protective layer.

Except for Ti and Zr oxides, we suppose that other metal oxides such as MgO, ZnO, and Al_2_O_3_ also have the ability to work as protective layer because these high-voltage metal oxides all have high tolerance for moisture.

### Cu^2+^ Substitution

The Cu^2+^ substitution is the simplest way to obtain air-stable Na_*x*_TMO_2_ compounds. The success of Cu^2+^ substitution to achieve high stability against moisture has been proven by many reports (Li Y. et al., 2015; Mu et al., 2015; Yao et al., 2017; Zheng L. et al., 2017; Chen et al., 2018; Deng et al., 2018), few references give the working mechanisms of Cu^2+^ in these air-stable Na_*x*_TMO_2_ compounds.

**Figure 12:**
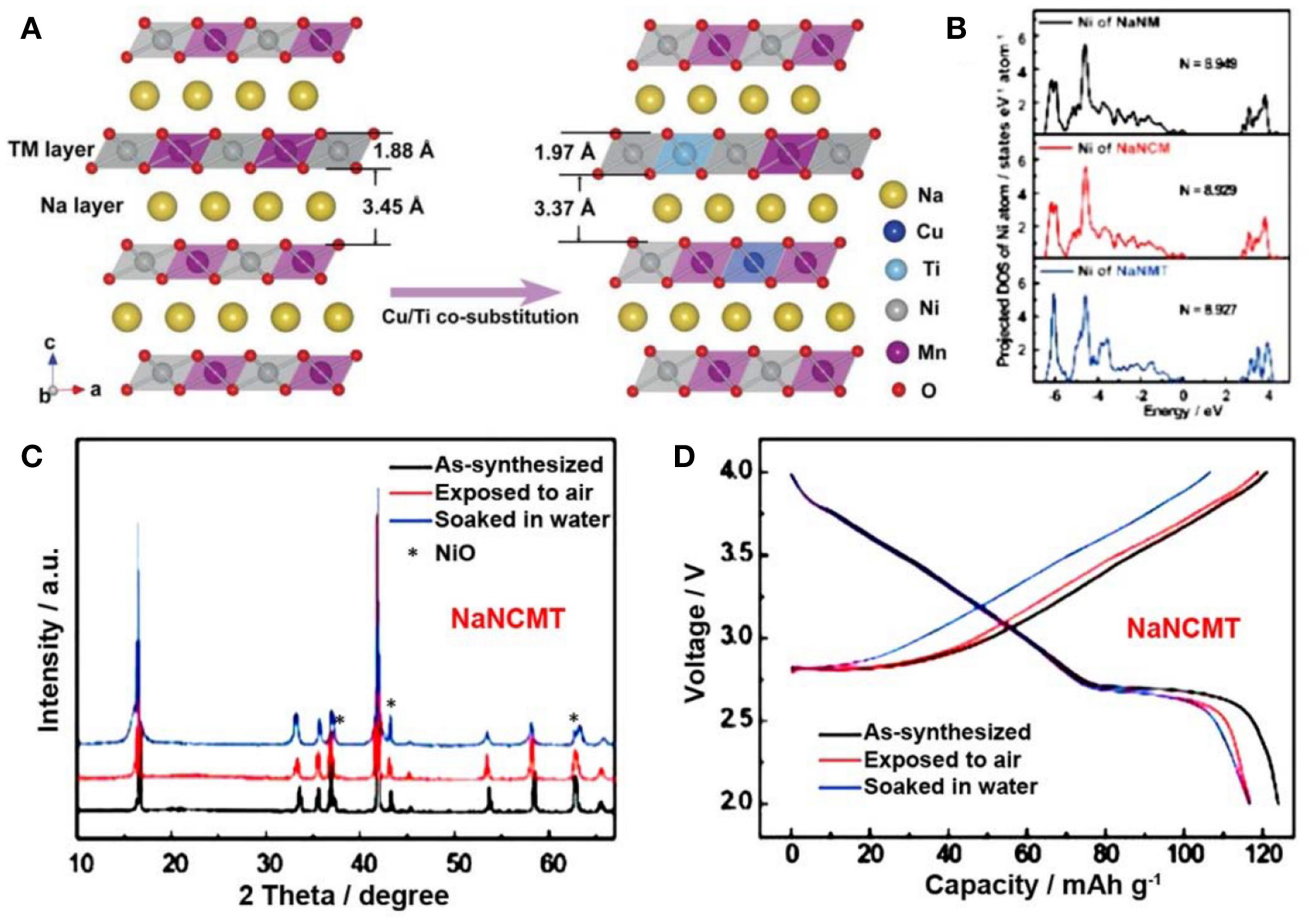
**(A)** refined crystal structure of NaNM and NaNCMT samples, **(B)** Electronic density of states on Ni ion of NaNM, NaNCM, and NaNMT samples, respectively. **(C)** XRD patterns of pristine, air-exposure and water soaking NaNCMT samples. **(D)** charge/discharge curves of pristine, air-exposure and water soaking NaNCMT samples. Reproduced with permission (Yao et al., 2017). Copyright 2017, American Chemical Society.

In, 2017, Yao et al. designed an air-stable O3-NaNi_0.45_Cu_0.05_Mn_0.4_Ti_0.1_O_2_ (NaNCMT) cathode though cosubstitution of Cu^2+^ and Ti^4+^ in O3-NaNi_0.5_Mn_0.5_O_2_ (NaNM) compound. This strategy could decrease the Na^+^ interlayer distance and increase the valence state of TM ions. According to the refined crystal structure of NaNM and NaNCMT, the interlayer distance was reduced from 3.45 Å to 3.37 Å, respectively (Figure 12A), which was in favor of preventing the insertion of water molecules. DFT calculation revealed that Cu/Ti cosubstitution facilitated the increasing in valence state of Ni (Figure 12B). Compared with NaNM compound (Figure 6B), the XRD pattern of NaNCMT sample showed no obvious peaks change after air-exposure or water soaking (Figure 12C). During charge/discharge process, only slight capacity decay was observed after aging experiments (Figure 12D). (Yao et al., 2017) However, the explanation about the relationship between valence state of TM ions and air-stability was not mentioned.

**Figure 13:**
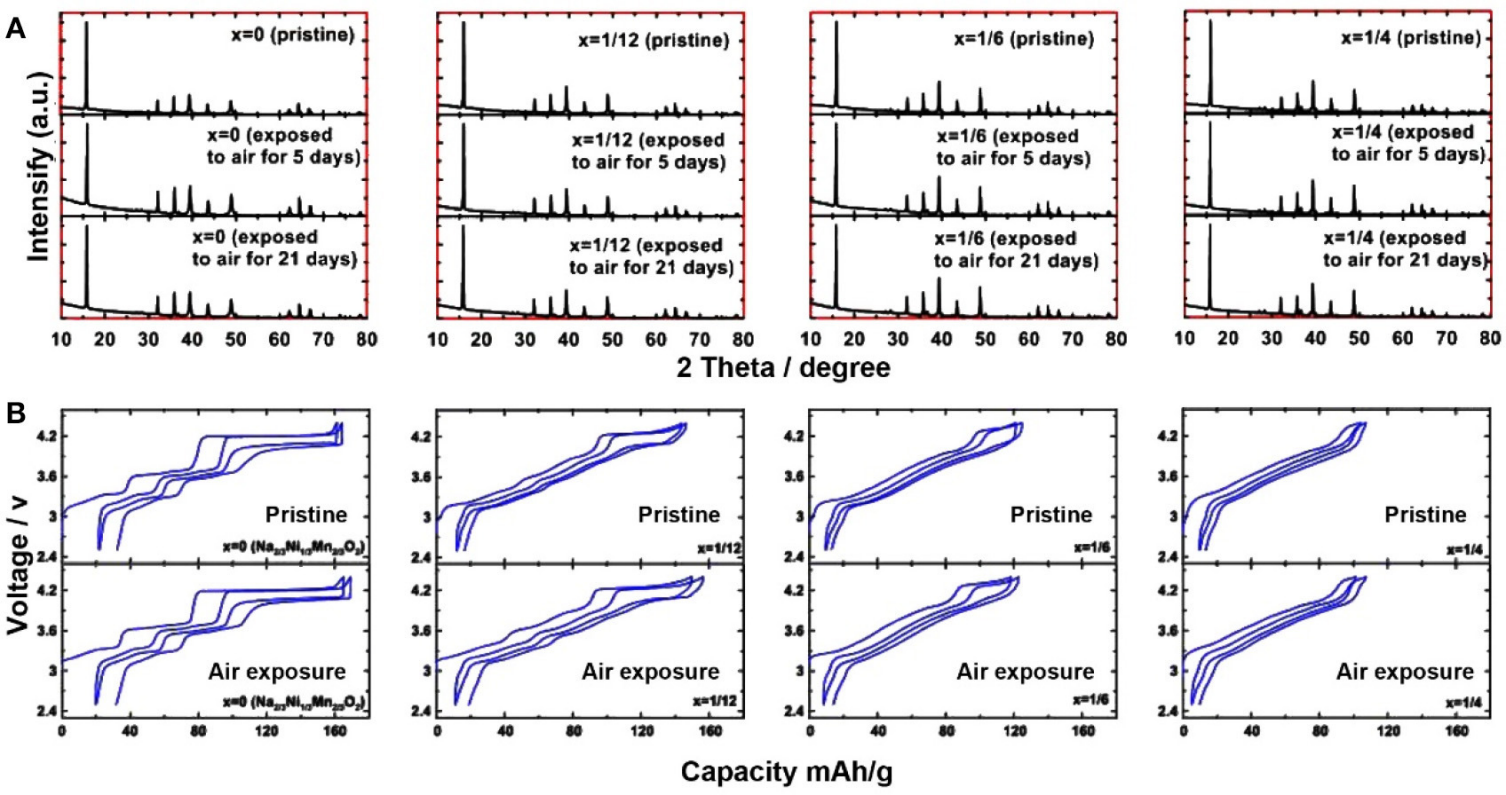
**(A)** XRD patterns of Na_2/3_Cu_*x*_Ni_1/3−x_Mn_2/3_O_2_ samples (*x* = 0, 1/12, 1/6 and 1/4) before/after air-exposure, **(B)** the corresponding charge/discharge profiles. Reproduced with permission (Zheng L. et al., 2017). Copyright 2017, American Chemical Society.

Zheng et al. investigated the structure stability of Na_2/3_Cu_*x*_Ni_1/3−x_Mn_2/3_O_2_ compounds (0 ≤ *x* ≤ 1/4) by air-exposure treatment. Compared to the XRD patterns of pristine samples, neither peaks position change nor new peaks formation were observed after exposing Na_2/3_Cu_*x*_Ni_1/3−x_Mn_2/3_O_2_ in air condition for 21 days (Figure 13A). According to charge/discharge profiles, all exposed electrodes had a little higher open circuit voltage than the un-exposed electrodes, but the average voltage and reversible capacity of the exposed electrodes showed no change or decay, indicating the air stability of these electrodes (Figure 13B). Since the radii of Cu^2+^ (0.73 Å) and Ni^2+^ (0.69 Å) were similar, replacing Ni^2+^ in Na_2/3_Ni_1/3_Mn_2/3_O_2_ by Cu^2+^ had no influence on the Ni/Mn cationic ordering arrangement. Therefore, the existence of Cu/Ni/Mn ordering arrangement could prevent the insertion of water molecules into the Na_2/3_Cu_*x*_Ni_1/3−x_Mn_2/3_O_2_ interlayer spacing because of the interlayer interaction between the adjacent Cu/Ni/Mn layer. However, no evidence was provided to prove the Cu/Ni/Mn ordering arrangement.

Other compounds such as O3-Na_0.9_Cu_0.22_Fe_0.30_Mn_0.48_O_2_, P2-Na_7/9_Cu_2/9_Fe_1/9_Mn_2/3_O_2_, O3-NaLi_0.05_Mn_0.5_Ni_0.3_Cu_0.1_Mg_0.05_O_2_, and P2-Na_0.6_Mn_0.9_Cu_0.1_O_2_ have been proved to be air-stable because all of their XRD patterns remained unchanged after air-exposure and water soaking treatment (Li Y. et al., 2015; Mu et al., 2015; Chen et al., 2018; Deng et al., 2018).

It seems that the Cu^2+^ plays an important role in maintaining the structure stability of these compounds under moisture. The reported Cu^2+^ substituted Na_*x*_TMO_2_ compounds, such as O3-NaNi_0.45_Cu_0.05_Mn_0.4_Ti_0.1_O_2_, P2-Na_2/3_Cu_*x*_Ni_1/3−x_Mn_2/3_O_2_, O3-Na_0.9_Cu_0.22_Fe_0.30_Mn_0.48_O_2_, O3-NaLi_0.05_Mn_0.5_Ni_0.3_Cu_0.1_Mg_0.05_O_2_, P2-Na_7/9_Cu_2/9_Fe_1/9_Mn_2/3_ O_2_, and P2-Na_0.6_Mn_0.9_Cu_0.1_O_2_, all show excellent structure stability under moisture condition. However, few investigations explain the working mechanism of Cu^2+^ substitution in these air-stable compounds. In O3-NaNi_0.45_Cu_0.05_Mn_0.4_Ti_0.1_O_2_ compound system, the working mechanism of Cu^2+^ is attributed to the increase of the Ni valence state by DFT calculation, but the relationship between valence state of Ni and air-stability is not clear so far. In addition, how to explain the Ni free compound systems for their air stability after Cu^2+^ substitution still remains problems.

Table 2 lists most of the air-stable Na_*x*_TMO_2_ compounds published to date, including the design strategies and the corresponding electrochemical performance.

**Table 2:** A summary of current air-stable Na_*x*_TMO_2_ cathode.

**Air stable materials**	**Design strategies**	**Test conditions**	**Rate performance**			**Cycling performance**			**References**
			**Maximum rate (*C*)**	**Capacity (mAh g^**−1**^)**	**Capacity retention**	**Cycle rate (*C*)**	**Cycle number**	**Capacity retention**	
Na_2/3_Ni_1/3_Mn_2/3_O_2_	Ni/Mn ordering arrangement	Air-exposure and water soaking	None	None	None	None	None	None	Lu and Dahn, 2001b
NaMnNi_0.1_Ti_0.1_O_2_	titanium (III) oxides protective layer	Air-exposure	10	102	55%	5	500	81%	Guo et al., 2017
ZrO_2_@NaNi_0.7_Mn_0.15_Co_0.15_O_2_	ZrO_2_ protective layer	Air-exposure	10	41	37%	0.5	200	70%	You et al., 2018
NaNi_0.45_Cu_0.05_Mn_0.4_Ti_0.1_O_2_	Cu/Ti co-substitution	Air-exposure and water soaking	4	100	80%	1	500	70.2%	Yao et al., 2017
Na_2/3_Cu*_*x*_*Ni_1/3−x_Mn_2/3_O_2_	Cu substitution	Air-exposure	5	80	87%	None	None	None	Zheng L. et al., 2017
Na_7/9_Cu_2/9_Fe_1/9_Mn_2/3_O_2_	Cu substitution	Air-exposure and water soaking	2	50	52%	1	150	87%	Li Y. et al., 2015
Na_0.9_Cu_0.22_Fe_0.30_Mn_0.48_O_2_	Cu substitution	Air-exposure	5	60	60%	0.1	100	97%	Mu et al., 2015
NaLi_0.05_Mn_0.5_Ni_0.3_Cu_0.1_Mg_0.05_O_2_	Cu substitution	Water soaking	1	125	71%	1	400	67.4%	Deng et al., 2018
Na_0.6_Mn_0.9_Cu_0.1_O_2_	Cu substitution	Air-exposure and water soaking	8	85	50.4%	4	250	70.4%	Chen et al., 2018

## Summary and Prospects

Air-stability is one of the key issues for practical application of Na_*x*_TMO_2_ SIB cathode materials. In recent years, with understanding the structure of Na_*x*_TMO_2_, several strategies have been developed to obtain air-stable Na_*x*_TMO_2_ compounds, including constructing TM ordering arrangement, coating protective layer and Cu^2+^ substitution. However, there still remain some challenges. For example, the reaction mechanism of the “strong interlayer interaction” for TM ordering arrangement as well as the substitution of Cu^2+^ and other cations should be further understood. In any case, we believe that the air-stable Na_*x*_TMO_2_ materials with low cost and high theoretical capacity are highly competitive as SIB cathode materials in the large-scale energy storage application.

## Author Contributions

RZ contributed conception and design of the manuscript. YZ organized the reference and wrote the first draft of the manuscript. All authors contributed to manuscript revision, read and approved the submitted version.

### Conflict of Interest Statement

The authors declare that the research was conducted in the absence of any commercial or financial relationships that could be construed as a potential conflict of interest.
